# Prevalence of burnout among healthcare professionals: a survey at fort portal regional referral hospital

**DOI:** 10.1038/s44184-024-00061-2

**Published:** 2024-05-06

**Authors:** Ian Batanda

**Affiliations:** Fort Portal Regional Referral Hospital, Fort Portal, Uganda

**Keywords:** Health care, Health occupations, Risk factors

## Abstract

The work environment in most hospitals is characterised by activities that are strenuous both physically and mentally. These can result in physical and mental exhaustion, which can lead to burnout if not adequately addressed. Burnout among healthcare professionals can negatively affect their clinical decision-making, quality of communication with patients and colleagues as well as their ability to cope with work-related pressure, and ultimately affect the quality of care and patient outcomes. The inclusion of burnout in the 11th revision of the International Classification of Diseases (ICD-11) as an occupational phenomenon indicates that it is an issue of concern in the workplace for which people may need professional attention. This descriptive cross-sectional survey aimed to determine the point prevalence of burnout among healthcare professionals at Fort Portal Regional Referral Hospital and the factors contributing to burnout. The study also evaluated the linear relationship between the age of workers, their work duration at the hospital, and their burnout score, in addition to the possible impact on patient care. Participants were selected from the hospital WhatsApp group and invitations to participate were sent to their individual accounts. Burnout was assessed using the Copenhagen Burnout Inventory. Generally, burnout scores ranged from 16% to 86%, with an overall mean burnout score of 57.4%. The notable factors contributing to burnout included imbalances in duty allocation, physically strenuous work, and resource constraints. Burnout of varying levels was found to be prevalent across all carders in the hospital, although the results indicate that most healthcare professionals experience moderate burnout. Most of the factors contributing to burnout are within the scope of hospital leadership to address. The possible impact on staff performance and patients’ clinical outcomes is speculative, and additional studies are required.

## Introduction

The healthcare work environment is characterised by strenuous activities^1^ which can predispose healthcare professionals to burnout, and negatively affect their work performance. This is made worse by a heavy workload which is a major problem for many healthcare systems with significant adverse effects on efficiency and patient safety^2^.

In most developing countries, the work environment in healthcare facilities is characterized by understaffing, excessive workload, and highly demanding tasks which undermines access to and provision of quality health services^3^. In Africa, for example, the ratio of 1.55 health workers (physicians, nurses and midwives) per 1000 people is below the WHO threshold density of 4.45 health workers per 1000 people needed to deliver essential health services^3,4^. While most developed countries have better health worker-to-patient ratios, understaffing is still a challenge. In Europe For example, the shortfall of health workers was estimated at 1.6 million in 2013 and is predicted to grow to 4.1 million by 2030^5^.

These staffing shortfalls expose health professionals to overwhelming workloads which increase the risks of stress, illness, and absenteeism^5^. The resulting physical and mental exhaustion can affect the worker’s clinical decision-making, quality of communication with patients and colleagues as well as the ability to cope with work-related pressure^6^. While burnout among health professionals and its impact on healthcare systems has been studied historically, it is not extensively documented in the African context where work in healthcare settings is often characterized by understaffing, resource limitations and excessive workload, which are known risk factors for work-related stress.

In the context of healthcare, studies on burnout emerged in the late 1960s to describe the emotional and psychological stress experienced by clinic staff caring for structurally vulnerable patients in free clinics. Over time the scope of burnout has evolved to include job-related stress in any health practice environment^6^. However, variations in definition and assessment methods make it difficult to standardize assessment within the hospital environment.

A systematic review of the prevalence of burnout among physicians found substantial variability in prevalence estimates of burnout among practising physicians and marked variation in burnout definitions, and assessment methods^6^. Another systematic review identified 88 unique definitions of burnout^7^. The marked variation in burnout definitions, assessment methods, and study quality highlights the need for developing a consensus definition of burnout and standardizing measurement tools to assess the effects of chronic occupational stress on healthcare professionals.

Attempts to expound the definition of burnout have broken it down into three components; personal burnout, work-related burnout, and client-related burnout^8^.

According to Kristensen et al., each component is explained as follows:

1. “*Personal burnout is the degree of physical and psychological fatigue and exhaustion experienced by the person*”.
2. “*Work-related burnout is “The degree of physical and psychological fatigue and exhaustion that is perceived by the person as related to his or her work*”.
3. “*Client-related burnout is the degree of physical and psychological fatigue and exhaustion that is perceived by the person as related to his or her work with clients*”.

These components enable the assessment of burnout from different perspectives and can be used to shed light on the possible factors contributing to overall burnout^8^. They enable attribution of an individual’s symptoms to their work, and the extent to which they see a connection between their fatigue and their work, regardless of their age, gender, or professional status, all of which can influence their perception of exhaustion^8^.

Recent attempts at harmonizing the definition of burnout have resulted in a simplified version which is believed to respond to the fundamentals of definition formation^7^. The definition is as follows: “*In a worker, occupational burnout or occupational physical AND emotional exhaustion state is an exhaustion due to prolonged exposure to work-related problems”*^7^

Furthermore, the WHO describes burnout as a syndrome characterized by three dimensions: feelings of energy depletion or exhaustion, increased mental distance from one’s job, feelings of negativism related to one’s job, and reduced professional efficiency resulting from chronic workplace stress that has not been successfully managed^9,10^. Although not classified as a mental disorder, burnout is still regarded as one of the reasons for which people may seek health services and is included in the 11th revision of the international classification of diseases (ICD-11) as an occupational phenomenon^9,11^. Its inclusion in the ICD-11 is an acknowledgement that burnout is an occurrence of concern at the workplace.

The lack of standard tools for assessing and documenting burnout means that many organizations including hospitals which are expected to be the custodians of health promotion do not assess burnout among their staff. It, therefore, remains undocumented, and its burden on the staff and the overall impact on service delivery remain a subject of speculation. Standardization of the definition and assessment methods of burnout is necessary if the assessment of burnout among healthcare professionals is to be integrated into the healthcare work environment to support health professionals.

With the evolution of the general view of burnout, several studies have been conducted among health professionals. Burnout syndrome has been reported among health professionals across all stages of their careers, with symptoms of emotional exhaustion and depersonalization considerably prevalent particularly among nursing staff. One study among primary healthcare professionals found a high risk for burnout syndrome in 10.6% of the participants. The study also found a 29.8% prevalence estimate of high-level symptoms of emotional exhaustion, and 22.3% of depersonalization^12^. The symptoms of burnout observed among healthcare professionals include emotional exhaustion, the dehumanization of interpersonal relationships, and loss of motivation or loss of self-fulfilment^13^. Dehumanization refers to the loss of one’s sense of what it means to be human and may be characterised by lacking emotions, warmth, and flexibility as well as treating patients and colleagues as immature, unintelligent, uncivilized, or irrational^14,15^. Other common symptoms include chronic exhaustion, reduced performance, and alienation from work activities^16^.

Among health professionals symptoms of burnout result from regular exposure to emotionally draining situations such as caring for patients with distressing symptoms, fatalities, as well as resource constraints that make certain patient treatments inaccessible^17^. Working in such a stressful environment for long periods with little time for recovery impacts the mental health of healthcare professionals and is a risk factor for burnout^18^.

Studies show that exposure to emotionally draining situations like caring for a high proportion of elderly patients and exposure to high fatality rates in inpatient units is associated with high levels of stress and emotional exhaustion among nurses^17,19^.

A recent study among nurses and student nurses at FPRRH examined the relationship between direct exposure to potentially traumatic events and symptoms of burnout. The results indicated that exhaustion symptoms of burnout are associated with secondary traumatic stress^20^. Although this study did not estimate the extent of emotional exhaustion and burnout among the staff at the hospital, it highlighted its presence among the nurses at FPRRH and informed the need for further research.

Work-related stress is particularly prevalent among medical trainees. In a study evaluating moral distress and burnout in internal medicine residents, 21% reported experiencing a high level of burnout^21^. Female residents in particular reported experiencing high levels of emotional exhaustion. Burnout rates for medical residents ranged from 27% to 75% across various medical subspecialties, with the highest reported levels among obstetrics-gynecology trainees and the lowest levels among family medicine trainees^21^.

However, health professionals at various career stages also experience considerable levels of burnout. One study suggests that emergency physicians experience the highest levels of burnout with 57% of emergency physicians experiencing burnout^22^. In a systematic review of burnout among physicians, the prevalence estimates of overall burnout ranged from 0% to 80.5%. These estimates were reported by 67.0% (122/182) of the studies. The prevalence estimates of emotional exhaustion ranged from 0% to 86.2% for Maslach Burnout Inventory (MBI)-derived emotional exhaustion. These were reported by 72.0% (131/182) of the studies included in the review. In addition, the review found that the prevalence estimates of a diminished sense of personal accomplishment ranged from 0% to 87.1%, as reported by 63.2% (115/182) of the studies^6^.

Burnout among the staff at FPRRH has previously been highlighted by a report of the auditor general on the financial statements of FRRH for the year ended 30th June 2016. The report acknowledged understaffing at the hospital and indicated that burnout of health workers was a possible consequence^23^. The report, which indicated a staffing rate of 76%, suggests that understaffing potentially overstretches the available staff beyond their capacity and negatively affects the quality of service delivery to the patients^23^.

Unfortunately, hospitals often lack adequate avenues for health professionals to report and address work-related stress and to manage the consequent burnout which results in increased staff turnover^24^. The individual healthcare worker often has the responsibility to recognize and manage their own stress and symptoms of burnout, with few avenues or tools institutionally available to assist them^18^. Mental health conversations in hospital work environments are also usually informal, yet if encouraged at an institutional level, and not stigmatised could help in minimizing burnout among healthcare professionals^25,26^. Additionally, healthcare workers have poor healthcare-seeking behaviour, particularly mental health support. A study to investigate the prevalence of mental help-seeking among public health workers, during covid 19 outbreak found that only 12.7% reported professional mental health-seeking^27^. Therefore, burnout among health workers is often undocumented and underreported.

Several factors have previously been identified as contributing to or worsening physical and mental exhaustion among health professionals, thereby leading to burnout.

A systematic review of burnout about specific contributing factors among nurses identified several factors and confirmed the relationship between work-related stress and burnout. Work environment-related stressors such as poor peer relationships, poor nurse-patient relationships, lack of professional recognition or reward feedback clarity, and supervisor leadership style were related to one or more burnout dimensions^28^. The study also found that work content-related stressors such as nursing role, patient care, job demands, job complexity, work overload, and working overtime were also related to burnout. Additionally, nurses who reported inadequate communication with doctors about patients, as well as fear of not completing tasks, also reported high burnout^28^.

Other studies also suggest that burnout (including all three dimensions) may be associated with time of work shift although there is no consensus on whether day or night duties are more stressful for health professionals. Some studies indicate that burnout is most frequently associated with recurrent night duty among nurses, while others suggest that burnout is significantly higher among those working the day shift^28,29^.

Age and career demands have also been reported as potential contributing factors. One study found the distribution of burnout among community psychiatric nurses displayed two slow peaks: one for the 30 s age group; and the other for the 50 s age group^30^. Differences in burnout prevalence between psychiatric and control groups were most noticeable for nurses in the 30 s age group. Nurses in this age bracket often have high expectations and heavy work demands heaped on them by both superiors and subordinates. These levels of expectation and work demands may prove excessive, causing extreme mental and physical exhaustion. The burnout peak in the 50 s age group may be associated with reduced physiological functioning and the associated increasing development of illness^30^.

Another study points to prolonged stay in the same job as another possible contributing factor^31^. Staying too long in the same job without career progression can lead to long-term exposure to stressors. However, some health professionals believe that some level of work-related stress is unavoidable and acceptable^31^. The study found that most participants agreed that some degree and type of stress was acceptable and unavoidable in healthcare. Working night shifts and on public holidays, treating patients who are in pain, and sharing distressing moments with patients and caregivers were some of the acceptable stressors^31^.

The causes of burnout are complex, multifactorial and may be interconnected. For example, it is difficult to separate general life stressors and job-related stressors as these are often overlapping and interconnected^32^. The imbalance between the demands of the job, income, and family demands can be both personal life and job-related stressors. Additionally, the interpersonal relationship with patients and co-workers, including superiors, could either be a stressor or a protector. A positive and harmonious work relationship with co-workers can help handle stressors, while a negative relationship can exacerbate work-related stress^31,32^.

Burnout significantly affects the well-being of workers and their productivity. This effect can be transferred to clients directly or indirectly. While burnout has been well-studied among healthcare professionals, few studies have focused on its impact on the patients they serve.

It is suggested that overwhelming exhaustion can create feelings of cynicism, detachment from the job, and a sense of ineffectiveness among health workers^33^. A study on the impact of burnout on self-reported patient care among emergency physicians indicated that emergency physicians with high levels of burnout were more likely to report performing suboptimal care practices such as admitting or discharging patients early, not answering patients’ questions, not treating patients pain, ordering more tests, not communicating important handoffs, and not discussing plans with other colleagues^22^. A qualitative study among practising general practitioners (GPs) indicated that the GPs believed that poor well-being and burnout affect the quality of care patients receive by reducing doctors’ abilities to empathize, reducing the ability to display positive attitudes and listening skills, and increasing the number of inappropriate referrals^34^. Another study also concluded that poor relations with patients, difficulty meeting patients’ needs, and high workload are all associated with burnout^28^. This is in line with the findings of a study among nurses which indicated that all burnout dimensions of the Copenhagen Burnout Inventory (CBI) were related to the outcome of patient safety grade and that healthcare organizations could reduce negative patient safety ratings by reducing nurse burnout^35^. The findings of these studies imply that in the hands of burnt-out health professionals the patient’s safety is considerably compromised, yet the patient is powerless about it.

In the presence of a high workload in a stressful environment, health professionals may struggle to maintain composure, which can affect their ability to listen to patients’ concerns and address them with empathy. This directly affects patient care and clinical outcomes. It can also affect their ability to communicate with kindness to colleagues and patients. The result is a toxic work environment with low staff morale and broken channels of communication between staff and patients, which can worsen stress and lead to poor patient outcomes and patient satisfaction.

Additionally, poor health and moderate to high levels of burnout among health professionals can result in increased medical errors and poorer patient safety. A survey among medical residents in Ireland showed levels of burnout correlated with an increase in medical errors. Sixty-four percent (64%) of the residents who experienced symptoms of burnout also reported making a medical error compared with 22% of those who did not experience symptoms^21^. Therefore, studies to evaluate the impact of health worker burnout on patient care are necessary.

The goal of the study was to determine the point prevalence of burnout among a sample population of health professionals at FPRRH. The study sought to estimate the extent of burnout among the health professionals at FPRRH, to identify workplace factors contributing to burnout at FPRRH and to examine the impact of burnout among health professionals on patient care.

The study examined the phenomenon of burnout in the healthcare work environment in the African setting and its impact on patient care and service delivery. It was expected to highlight the magnitude of the problem and present a basis for additional research into work-related stress among healthcare professionals. The results were expected to inform policy and hospital managers regarding implementing measures to address workplace stress. It was also expected to highlight the growing need for healthcare professionals to actively seek mental health support and improve staff welfare.

## Methods

### Study design and setting

The study was a descriptive cross-sectional survey to determine the point prevalence of burnout among healthcare professionals at Fort Portal Regional Referral Hospital (FPRRH) which serves the Rwenzori region of western Uganda. The region has a population of 2,868,000 people as of 2019. The population is expected to grow at a rate of 3% per year with a projected population of 3,355,437 by 2024^36^. The region is comprised of 8 districts namely, Kabarole, Kamwenge, Kasese, Ntoroko, Bundibugyo, Bunyangabu, Kyenjojo, and Kyegegwa districts as well as a regional city called Fort Portal Tourism City. The hospital was projected to have 34,000 admissions and 120,000 outpatients in 2021/2022 with a bed occupancy rate of 85%^37^. The hospital had an approved staff structure of 428 positions in 2016 although only 324 positions were filled, leaving 104 positions vacant^23^. The auditor general’s report details the staff as follows; 17 doctors (5%), 130 nurses (40%) and 62 allied health professionals (19%)^23^. This staffing structure had not changed significantly by 2022, although a new structure was in the plan for all regional referral hospitals.

### Study population

The study was conducted among health workers of different carders broadly categorized into the following groups: Doctors, Nurses, and Allied Health Professionals. All health professionals working as full-time employees at FPRRH were eligible for inclusion in the study. Employees with a total work duration of less than one year (recently recruited) were excluded.

The sample size was determined using the Krejcie and Morgan table. Population size (N) = 324. Sample size (S) = 175. A proportionate non-random sample of participants was computed for each group using the formula^38^:

$${Sample}\,{size}\,{of}\,{each}\,{layer}\,({\rm{s}})\,{\boldsymbol{=}}\,({size}\,{of}\,{layer}{\boldsymbol{/}}{size}\,{of}\,{population}){\bf{X}}({size}\,{of}\,{the}\,{whole}\,{sample}).$$

Therefore, the number of participants from each group was determined as follows: Doctors = (17/324) X175 = 9, Nurses = (130/324) X175 = 70, and Allied health professionals (62/324) X175 = 33.

### Study outcomes

The primary outcome was the prevalence of burnout among the health professionals at FPRRH. The secondary outcomes were factors contributing to burnout, impact on patient care, and a correlation between the age of the health workers, duration of work at FPRRH and burnout burden. The extent of burnout was measured using the Copenhagen burnout inventory (CBI). Data from client-related burnout was used to assess the impact of burnout on patient care.

### Data collection instruments

Data was collected using self-administered digital questionnaires prepared using Google Forms. The questionnaire incorporated questions from the Copenhagen Burnout Inventory (CBI) and followed the format of the CBI inventory. The CBI is a questionnaire with three sub-dimensions: Personal burnout, work-related burnout, and client-related burnout. This enabled the assessment of burnout from different perspectives^8^. The questionnaire which used multiple-choice questions included four sections namely; Participant demographics with 5 questions, Personal burnout with 6 questions, Work-related burnout with 12 questions, and Client-related burnout with 8 questions. The section on participant demographics captured data on age, sex, duration of work at FPRRH, and employment category.

### Data collection procedure

A convenience sample of healthcare professionals at FPRRH was selected from the hospital WhatsApp group and invitations to participate were sent to their individual accounts. A link to the data collection tool was sent to the participants’ individual WhatsApp accounts alongside the invitation to participate. The data collection tool was pre-tested before actual data collection to ensure usability. The participants were free to fill out and submit the questionnaire at their time of convenience. The completed questionnaires were then stratified into three broad categories namely, Doctors, Nurses, and Allied Health Professionals.

### Data management and analysis

The questionnaires were checked for completeness, the data was sorted and summarized using Google Sheets and Microsoft Excel (Microsoft 365 MSO (Version 2308). Each response was assigned a score as guided by the Copenhagen Burnout Inventory (CBI) as follows: Always: 100. Often: 75. Sometimes: 50. Seldom: 25. Never: 0. A score for each section of the data collection tool was computed as the average of the scores on the items. A total burnout score was computed as the average score from all three sections.

Burnout was categorized as low, moderate, and high burnout according to Kristensen’s criteria for burnout levels^39^. A score of 0-49% represented low burnout, 50-75% represented moderate burnout and ≥ 76% represented high burnout.

Descriptive statistics including percentages, means and standard deviations, as well as Pearson correlation coefficients, were computed using IBM SPSS statistical package version:29.0.0.0(241) as well as Microsoft Excel 365 MSO (Version 2308). 2-tailed 95% confidence intervals for the correlations and statistical significance were estimated based on Fisher’s r-to-z transformation with bias adjustment. The data was then presented in frequency charts and tables.

The factors contributing to burnout were identified from participant responses to specific questions in the CBI as part of the data collection questionnaire. Any of those questions was considered a potential contributing factor if the majority of the participants rated it 50 or higher on the Likert scale (To a very high degree: 100; To a high degree: 75; Somewhat: 50; To a low degree: 25; To a very low degree: 0)^40^. The impact of burnout on patient care was assessed by examining responses in the client-related burnout section of the CBI. Responses of those who scored low, moderate, or high burnout were compared in terms of frequencies.

### Ethical consideration

The study was reviewed by the Fort Portal Regional Referral Hospital Research and Ethics Committee (FPRRH-REC) and approved by the FPRRH administration. To ensure confidentiality, the questionnaires were anonymized, coded, and kept securely in a password-protected folder on Google Drive accessed only by the researcher^41^.

All participants were required to give written consent before participating in the study. The study was not expected to pose any physical or psychological risk to the participants. However, participation may have caused minimal disruption to their work routine.

## Results

Over 2 weeks in June 2022, A total of 31 (*n* = 31) healthcare professionals at Fort Portal Regional Referral Hospital participated in the study as per the inclusion criteria, representing an overall response rate of 28%. This response rate was acceptable for a web-based survey where no follow-up or incentives were provided, and sample representativeness was given primary importance^42^. Based on sample size estimates, the response rate for doctors was 44.4%, the response rate for nurses was 22.8%, and that for allied health professionals was 33.3%.

### Participant characteristics

Most of the participants, 19 (61.3%) were females, while 12 (38.7%) were males.

In terms of age, 12 (38.7%) participants were in the range of 40–49 years, 10 (32.3%) were in the range of 20–29 years, and 9 (29%) were in the age range of 30–39 years. There were no participants in the age group of 50 to 60 years. The majority, 16 (51.6%) were nurses, 11 (35.5%) were allied health professionals and 4 (12.9%) were doctors.

In terms of work duration, 12 (38.7%) participants had worked at FPRRH for more than 10 years, 9 (29%) had worked at the hospital for 4 to 6 years, 7 (22.6%) had worked for 1 to 3 years, while 3 participants (9.7%) had worked for 7 to 10 years. The mean duration of work at the hospital was 6.32 years (SD = 4.308) as shown in Table 1.

**Table 1:** Summary of demographic characteristics of the participants

Characteristics	Frequency	(*n* = 31)
Age group (years)		
20-29	10 (32.3%)	
30-39	9 (29%)	
40-49	12 (38.7%)	
50-60	0	
Median age	30 years	
Sex		
Male	12 (38.7%)	
Female	19 (61.3%)	
Duration of work at FPRRH (years)		
1-3	7 (22.6%)	
4-6	9 (29%)	
7-10	3 (9.7%)	
> 10	12 (38.7%)	
Mean duration	6.32 years (SD = 4.308)	
Employment Category		
Doctors	4 (12.9%)	
Allied health professionals	11 (35.5%)	
Nurses	16 (51.6%)	

### Extent of burnout among the health professionals at FPRRH

**Fig. 1:**
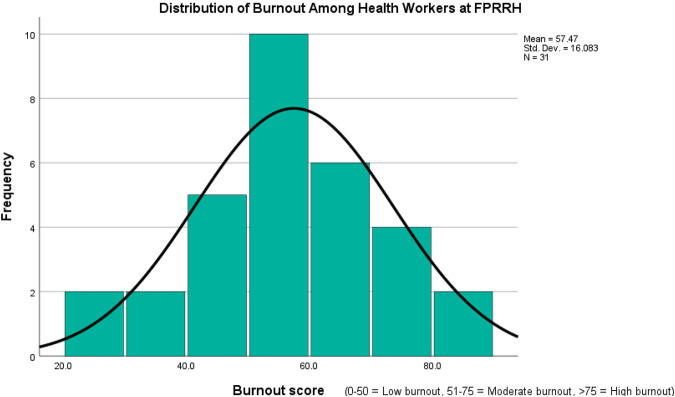
Distribution of burnout scores among staff at Fort Portal Regional Referral Hospital. Most of the staff scored moderate burnout.

Burnout scores ranged from 16% to 86%, with an overall mean burnout score of 57.4% (SD = 16.083). The distribution of overall burnout is shown in Fig. 1. The majority, 19 (61.2%) scored moderate burnout, 9 (29%) participants scored low burnout, while only 3 (9.6%) scored high burnout.

The male participants had an average burnout score of 59.94% with an average age of 28.33 years, while female participants had an average burnout score of 55.92% with an average age of 32.10 years. Participants in the age range 30–39 had the highest average burnout score compared to the other age groups, while those of 20–29 years had the lowest average burnout score as shown in Table 2.

**Table 2:** A: Average Burnout score by Average age and gender. B**:** Average Burnout score by age group

Gender	Average Burnout score (%)	Average Age (years)
A		
Male	59.94	28.33
Female	55.92	32.10
Total	57.47 (SD = 16.083)	30.64 (SD = 8.53)

B		
Age range (years)		Average Burnout score (%)
20-29		52.41
30-39		61.36
40-49		58.78

### Burnout rate by age and duration of work

Pearson correlation indicated a weak positive correlation between the age of the health workers and burnout score, r(29) = 0.16, *p* =0.387. (95% CI [-0.208,0.485]).

There was also a weak positive correlation between the duration of work at Fort Portal Regional Referral Hospital and burnout score, r(29) = 0.11, *p* = 0.955. (95% CI [−0.345,0.365]).

**Table 3:** Possible causes of burnout identified out of participant responses in the three components of the CBI

Cause	Majority Rating	Frequency
Unbalanced duty allocation	Somewhat	38.7%
Physically exhausting work	To a very high degree	35.5%
Emotional exhaustion	To a very high degree	32.3%
Getting blamed for other people’s mistakes	Somewhat	35.5%
Nothing to do for a patient	To a very high degree	58.1%

Several factors were identified as possible contributors to burnout in the healthcare workplace, as shown in Table 3.

Causes were selected based on majority rating.

### Burnout rate among doctors

**Fig. 2:**
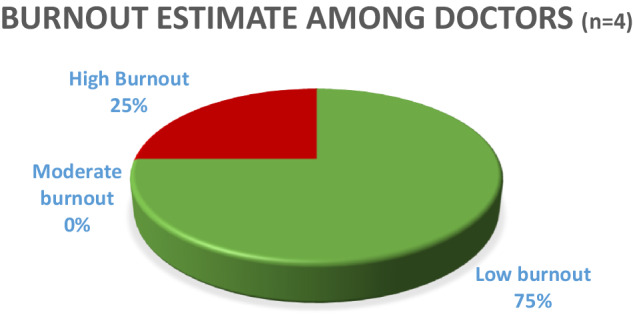
Estimates of burnout burden among doctors at FPRRH.

All doctors who participated in the study were male. Of these, 3 (75%) scored low burnout. Only 1 doctor (25%) had a score of high burnout as shown in Fig. 2. The doctor who scored high burnout was in the age range of 20–29 years, with a work duration of 1–3 years.

**Fig. 3:**
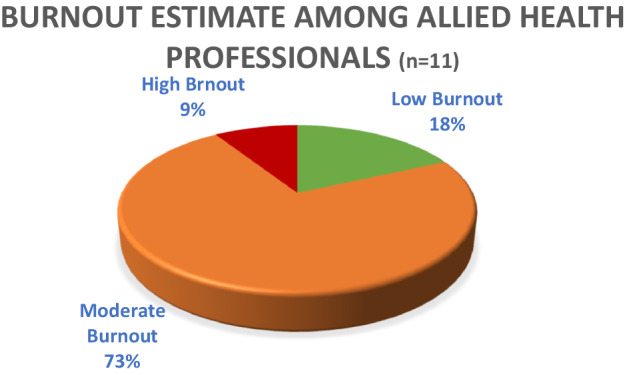
Estimate of burnout burden among allied health professionals.

**Fig. 4:**
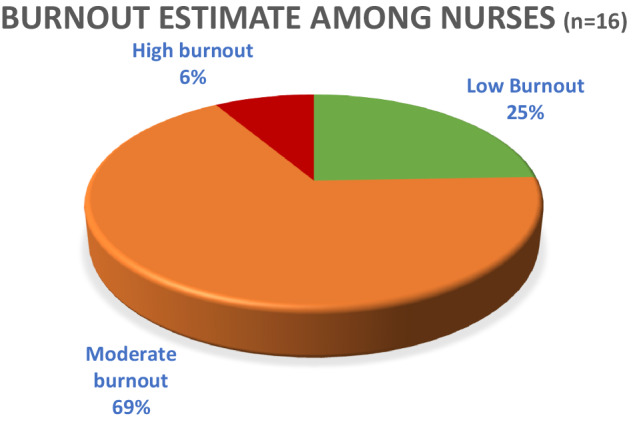
Estimate of burnout burden among nurses at FPRRH.

Of the doctors who scored low burnout 1 (33.3%) was in the age range of 20–29 years with 1–3 years of work duration. The other 2 (66.6%), were 40–49 years of age. Fifty percent (50%) of these had a work duration between 4 and 6 years, and the other 50% had worked for more than 10 years (Figs. 2–4).

Pearson correlation was computed to assess the linear relationship between doctors’ age, and burnout score, as well as doctors’ duration of work and burnout score. There was a strong negative correlation between doctors’ age and burnout score, r(2) = −0.66, *p* = 0.341. (95% CI [−0.990,0.856]). There was also a negative correlation between doctors’ duration of work at FPRRH and burnout score, r(2) = −0.58, *p* = 0.417. (95% CI [−0.987,0.883]).

### Burnout Rate among Allied Health Professionals

All allied health professionals who participated in the study were between 30 and 49 years of age with at least 4 years of work duration. Of these, 6 (55%) were 40–49 years of age, while 5 (45%) were between 30 and 39. The majority, 7 (63%) were females, and 4 (37%) were males.

Among the allied health professionals, 2 (18.1%) scored low burnout, 8 (72.7%) scored moderate burnout. Only 1 (9%) had high burnout as shown in Fig. 3. The allied health professionals who scored low burnout were all female, between 40 and 49 years of age. Fifty percent (50%) of them had worked at the hospital between 4 and 6 years, and the other 50% had worked longer than 10 years. The allied health professional who had high burnout was male in the age range of 40 to 49 and had worked for more than 10 years at the hospital.

Of the allied health professionals who scored moderate burnout, 3 (37.5%) were between 40 and 49 years of age, and 5 (62.5%) were between 30 and 39 years of age. Of these, 3 (37.5%) were male, while 5 (62.5%) were female.

Pearson correlation indicated a weak negative correlation between duration of work at FPRRH and burnout score, r(9) = −0.12, *p* = 0.718. (95% CI [−0.670,0.519]). There was a weak negative correlation between their age and burnout scorer (9) = −0.13, *p* = 0.695. (95% CI [−0.676,0.512]).

### Burnout rate among nurses

The nurses who participated in the survey were aged 20 to 49 years. The majority, 11 (68%) were females while 5 (32%) were males. Of these, only 1 nurse (6%) scored high burnout, 11 nurses (68.7%) scored moderate burnout, and 4 nurses (25%) scored low burnout as shown in Fig. 4. The nurse who scored high burnout was between 40 and 49 years of age and had worked at the hospital for longer than 10 years. Of those who scored moderate burnout, 3 (27.2%) were males while 8 (72.7%) were females. The majority, 5 (45.4%) were between 20 and 29 years of age, 3 (27.2%) were between 30 and 39 years and 3 (27.2%) were between 40 and 49 years of age. 4 (36.3%) had worked at the hospital between 1 and 3 years, 5 (45.4%) had worked between 4 and 10 years and 2 (18.1%) had worked for more than 10 years.

Of the nurses who scored low burnout 3 (75%) were females and 1 (25%) were male. All were in the age range of 20 to 39 years. Fifty percent (50%) of them had worked between 4 to 10 years, 25% percent had worked between 1-3 years, and the other 25% had worked more than 10 years.

A Pearson correlation coefficient was computed to assess the linear relationship between nurses’ duration of work at FPRRH and burnout score, as well as nurses’ age and burnout score. There was a weak positive correlation between the duration of work and burnout score, r(14) = 0.20, *p* =0.452. (95% CI [−0.332,0.630]). There was a statistically significant positive correlation between nurses’ age and burnout score r(14) = 0.56, *p* =0.025. (95% CI [0.067,0.819]).

### Workplace factors contributing to burnout

#### Unbalanced duty allocation

The majority, 15 (48.4%) of the participants indicated that duty allocation in their ward was somewhat frustrating. Four (4) of the participants (12.9%) indicated that duty allocation was frustrating to a high degree, and 4(12.9%) reported duty allocation was frustrating to a low degree. Fourteen (14) participants (45.2%) indicated that duty allocation in their ward needed to change for them to enjoy their time at work.

Of those who scored high burnout 1 (33.3%) reported that they always felt duty allocation on their ward needed to change for them to enjoy their time at work. 1 (33.3%) reported that they often felt duty allocation needed to change, and 1 (33%) reported that they sometimes felt duty allocation on their ward needed to change for them to enjoy their time at work.

In comparison, of those who scored moderate burnout, 10 (52.6%) indicated that they sometimes felt duty allocation on the ward needed to change for them to enjoy their time at work, 6 (31.5%) indicated that they always felt duty allocation needed to change, 1 (5.2%) indicated that they often felt duty allocation needed to change, 1 (5.2%) indicated that they seldom felt duty allocation needed to change and another 1 (5.2%) indicated that they felt duty allocation never needed to change for them to enjoy their time at work.

### Physically exhausting procedures

Another factor identified was physical exhaustion from medical procedures, with 11 (35.5%) of all the participants indicating that their work was physically exhausting to a very high degree. Ten (10) participants (32.3%) indicated that work was physically exhausting to a high degree, and 3 (9.7%) said it was exhausting to a low degree. Only 1 (3.2%) reported that their work was physically exhausting to a very low degree.

Of those who scored high burnout, 2 (66.6%) reported that their work was physically exhausting to a very high degree while the other 1 (33.3)% felt their work was physically exhausting to a high degree.

In comparison, of those who scored moderate burnout, 8 (42.1%) indicated that their work was physically exhausting to a very high degree, 7 (36.8%) indicated that their work was physically exhausting to a high degree, 3 (15.7%) indicated that their work was somewhat physically exhausting, and 1 (5.2%) indicated that their work was physically exhausting to a low degree.

### Emotional exhaustion

The other factor was emotional exhaustion associated with emotional attachment to patients. Seventeen (17) participants (54%) reported that they were emotionally attached to patients to a high degree. Five (5) participants (16.1%) indicated they get emotionally attached to patients to a very high degree, while 6 (19.4%) reported they somewhat get emotionally attached to patients.

Ten (10) participants (32.3%) indicated that they found their work to be emotionally exhausting to a very high degree, and another 10 (32.3%) reported that work was emotionally exhausting to a high degree. Seven (7) participants (22.6%) indicated their work was somewhat emotionally exhausting, while 4 (12.9%) indicated that it was exhausting to a low degree.

Of those who scored moderate burnout, 9 (47.4%) indicated that their work was emotionally exhausting to a high degree. Seven (7) participants (36.8) indicated that their work was emotionally exhausting to a very high degree, and 3 (15.8%) indicated that their work was somewhat emotionally exhausting. In contrast, all those who scored high burnout 3 (100%) indicated that their work was emotionally exhausting to a very high degree.

### Getting blamed for other people’s mistakes

Eleven (11) participants (35.5%) reported that they sometimes get blamed for other people’s mistakes. Another 8 (25.8%) indicated that they get blamed for other people’s mistakes often. Additionally, 4 (12.9%) indicated that they seldom get blamed for other people’s mistakes, and another 4 (12.9%) indicated never get blamed for other people’s mistakes. Only 2(6.5%) indicated that they always get blamed for other people’s mistakes.

### Resource limitations

Limitation in patient care was also identified as a potential factor. The majority, 18 (58.1%) of the participants reported that finding that there isn’t much they can do for a patient was frustrating to a very high degree. And, 6 (19.4%) indicated that it was frustrating to a high degree. Four (4) participants (12.9%) reported that it was somewhat frustrating, while 3 (9.7%) reported that it was frustrating to a low degree.

Of those who scored moderate burnout, 12 (63.1%) reported that finding that there isn’t much they can do for a patient was frustrating to a very high degree, 4 (21%) found it frustrating to a high degree, 2 (10.5%) found it somewhat frustrating, and 1 (5.2%) found it frustrating to a low degree. In contrast, all those who scored high burnout (100%) reported that finding that there isn’t much they can do for a patient was frustrating to a very high degree.

### Impact on patient care

Regarding the impact on patient care, 14 (45.2%) participants indicated that they somewhat found it hard to work with patients, 4 (12.9%) indicated that they found it hard to work with patients to a high degree, 7 (22.6%) indicated that they found it hard to work with patients to a low degree, while 5 (16.1%) reported that they found it hard to work with patients to a very low degree.

Of those who scored moderate burnout, 15 (78.9%) reported that their work with patients was somewhat frustrating. Only 2 (10.4%) indicated that it was frustrating to a high degree or a very high degree. And 1(5.2%) indicated that it was frustrating to a low degree. Twelve (12) participants (63.1%) of those who scored moderate burnout indicated that they felt they were sometimes tired of working with patients. 2 (10.5%) reported they often felt they were tired of working with patients. 2 (10.5%) reported they never felt they were tired of working with patients, 1 (5.2%) always felt they were tired of working with patients, and 1 (5.2%) reported they seldom felt they were tired of working with patients.

In comparison, of the participants who scored high burnout, 2 (66.6%) indicated they sometimes found it frustrating to work with patients, and 1 (33.3%) reported frustration to a high degree. Of the participants who scored high burnout 2 (66.6%) indicated they often felt they were tired of working with patients. 1 (33.3%) reported they were sometimes tired of working with patients.

In contrast, 5 (55%) of the participants who scored low burnout indicated that it was frustrating to work with patients to a low degree, while for the other 4 (44.4%), it was frustrating to a very low degree. Six(6) of the participants (66.6%) who scored low burnout indicated that they never felt tired of working with patients, and the other 3 (33.3%) indicated that they rarely felt tired of working with patients.

## Discussion

The results of this survey are consistent with previous studies indicating that burnout is prevalent among health professionals, although its severity varies across different age groups and different categories of health professionals, namely, Doctors, Nurses, and Allied health professionals. This study is among the few that included allied health professionals and compared findings in the three categories of health workers. Although allied health professionals constitute a large percentage of staff in most hospitals globally, there is limited published data on burnout in this group with most of the studies focusing on doctors and nurses.

The findings of this study indicate that the majority of health professionals at FPRRH, regardless of category, experience moderate burnout with burnout scores ranging from 16% to 86%. This is consistent with other studies which found burnout scores among physicians to range from 0% to 80%, and that the prevalence of burnout and its related problems was high among allied healthcare staff^43,44^. Similarly, a systematic review of estimates of burnout revealed prevalence estimates of overall burnout reported by 67.0% (122/182) of studies that provided data on overall burnout to range from 0% to 80.5%^6^.

This study found males to have a higher average burnout score, compared to females which is in contrast with previous studies which found that female health professionals had higher burnout scores possibly because women have disproportionate responsibilities outside work^21,45^.

The positive correlation between the age of the health workers and burnout score suggests that generally, as health professionals grow older, they are more likely to experience higher burnout scores. Similarly, the positive correlation between duration of work at FPRRH and burnout score, also suggests that the longer the health care professionals work, the more likely they are to experience burnout. However, these correlations were not statistically significant, and analysis of each category of health professionals separately showed somewhat contradictory findings for doctors and allied health professionals in terms of the linear relationship between their age, their duration of work, and burnout scores.

Among doctors, this study found low burnout was most prevalent (75%). This contrasts the findings of a systematic review among health care providers in sub-Saharan Africa which concluded that high burnout was prevalent among doctors^46^. The strong negative correlation between doctors’ age and burnout score suggests that as doctors grow older, they experience less burnout. The negative correlation between doctors’ duration of work at FPRRH and burnout score also suggests that doctors who have worked longer experience less burnout. This finding is consistent with previous studies showing higher burnout rates among residents and junior doctors^21,47^. This could suggest that older doctors have developed better mechanisms of coping with work-related stress. Alternatively, it could indicate that the younger doctors bear most of the workload compared to older doctors. The 25% rate of high burnout among doctors in this study was among younger doctors with shorter duration of work and is consistent with the 21% rate of high burnout among internal medicine residents found in an earlier survey^21^.

Similarly, among the allied health professionals, the negative correlation between duration of work at FPRRH and burnout score, suggests that allied health professionals who have worked longer at the hospital experience less burnout. The negative correlation between age and burnout score also suggests that older allied health professionals experience less burnout.

In contrast, among the nurses, the statistically significant positive correlation between nurses’ age and burnout score indicates that burnout is likely to increase with age. The positive correlation between the duration of work at FPRRH and burnout score also indicates that nurses who have worked longer at the hospital experience more burnout. This finding is consistent with previous studies showing significant levels of burnout among nurses^48,49^. One study found the distribution of burnout among community psychiatric nurses displayed two slow peaks: one for the 30 s age group; and the other for the 50 s age group suggesting that age and career demands can contribute to burnout^30^.

This contrast between nurses and the other categories of health workers could indicate that factors associated with burnout are more prevalent among nurses. Nurses bear a big portion of workload in patient care and various studies report imbalances in workload as a significant factor contributing to burnout among nurses^48,49^. Although this study did not examine workload for each category – which necessitates additional studies – the findings are consistent with previous studies that indicate higher burnout rates among nurses in comparison to other categories of health professionals^10,46^.

Burnout is a multifactorial phenomenon whose causes can involve individual, interpersonal and organizational stressors^50^. This survey, however, focused on identifying workplace factors potentially contributing to burnout among health professionals. Several factors were identified.

A significant proportion of participants indicated that duty allocation on their ward needed to change for them to enjoy their time at work. This is consistent with the findings of other studies identifying workload imbalances as a source of work-related stress^48,49,51^. Careful allocation of duties within hospitals is critical for balancing workloads and together with adequate staffing may lower distress and improve work experience^44,46^, which can minimize stress and improve staff performance and efficiency in the functioning of health care systems.

However, balancing the workload equitably is not always possible and can be challenging in cases of inadequate staffing versus workload. Other factors like illness, maternity leave, and absenteeism affect the distribution of workload among the staff, which can lead to burnout among those available for work. Healthcare managers need to address staffing levels, as well as absenteeism of health professionals in hospitals, to achieve the recommended patient-staff ratios^10^. Further studies are needed to obtain an in-depth understanding of duty allocation and workload imbalances.

Many of the participants indicated that their work was physically exhausting to a high degree or a very high degree. Most of the tasks involved in patient care are physically strenuous, yet they must be done routinely. Over time, this can result in physical exhaustion and negatively affect the physical and mental health of health professionals^24,52^.

The majority (64.6%) of the participants in this study reported that their work was emotionally exhausting to a high degree or a very high degree of CBI-derived work-related burnout. This is consistent with previous prevalence estimates of emotional exhaustion reported by 72.0% (131/182) studies which showed scores ranging from 0% to 86.2% for Maslach burnout inventory (MBI)-derived emotional exhaustion^6^. Work-related emotional exhaustion therefore has a significant association with burnout.

Health professionals are constantly exposed to highly emotional situations involving patients and caregivers, especially when the illness is chronic or life-limiting. In situations of lengthy hospital stays, some health workers tend to get attached to patients for several reasons such as having a previous experience with a certain illness or circumstance. In this study, 54.8% of the participants indicated they get emotionally attached to patients to a high degree. This is similar to a study which concluded that physicians frequently experience intense emotions when dealing with patients and that the emotions can be long-lasting and affect the patient-physician relationship^53^. The study also found that most physicians tried to control their reactions, and coping strategies included behavioural and cognitive approaches such as touching, smiling, crying, etc.

Most of the participants reported that finding that there isn’t much they can do for a patient was frustrating to a very high degree. Having little to do for patients can result from resource limitations, especially in a low-resource setting. Resource limitations can include drug and sundries stockouts, a lack of appropriate investigation modalities and an absence of specialists to manage illnesses.

Like many hospitals in developing countries, Fort Portal Regional Referral Hospital is faced with resource constraints. For many patients, certain diagnostic and therapeutic interventions are absent and inaccessible. Such patients require referral to the national referral hospital but often can’t afford to travel. This can cause health professionals to feel like there isn’t much more they can do for the patient. When such patients accumulate on the wards, frustration can build up among the healthcare team. This is because such patients are perceived to occupy hospital beds with little to no progress in their clinical care, or clinical outcomes.

There is a considerable impact on patient care, as well as on the overall healthcare system. Burnout syndrome is characterized by negative attitudes towards work and life and reduced personal accomplishment^46^.

In this study, the majority of those who scored moderate burnout, which was most prevalent, reported that their work with patients was somewhat frustrating and that they were sometimes tired of working with patients. In contrast, participants who scored low burnout indicated that working with patients was frustrating to a low degree or to a very low degree. And that they rarely or never felt tired of working with patients.

These findings suggest that in comparison to those with low burnout, health professionals with moderate burnout can sometimes have a negative outlook on working with patients and could sometimes perceive it as a burden. This can negatively impact the quality of patient care in several ways.

For example, in times when they feel frustrated or tired of working with patients, communication with patients and colleagues may be less courteous. This can impair the doctor–patient relationship and create a barrier against patients expressing their concerns. This is supported by a recent study, which found that doctors with burnout were twice as likely to receive low satisfaction ratings from patients^47^. It is also in agreement with the suggestion that increasing levels of burnout among health professionals can increase medical errors and affect patient safety^21^.

Health professionals experiencing physical exhaustion and frustration with work may be less enthusiastic to take up elective or semi-elective physically demanding procedures. It can also hinder their willingness to offer extra care or support to the patients and caregivers. Previous studies indicate that physical and mental exhaustion impact health workers’ clinical decision-making, quality of communication with patients and colleagues, as well as the ability to cope with work-related pressure, and that emotional exhaustion contributes most to increases in the turnover intention of health professionals^6,54^.

Additionally, studies show that doctors with burnout are more likely to be involved in patient safety incidents^47,54^. Therefore, burnout among health professionals has a direct impact on patient care and needs to be effectively addressed to preserve patient safety and clinical outcomes.

Physically demanding work also causes physical health issues. For example, low back pain is common among health workers due to bending and lifting activities associated with patient care. This can affect longevity in care delivery and could contribute to a high turnover of medical professionals in practice^47,51,54^. It can also lead to increased employee absence and reduced productivity in the workplace, which negatively affects the organization’s overall performance.

Although it is difficult to quantify the impact of burnout on patient care and clinical outcomes, there is evidence that it can affect staff performance. Health care organizations therefore need to invest in mechanisms to address burnout among health professionals as a means of ensuring patient safety^47^.

The sudy had a number of limitations. It was restricted to FPRRH, and the small number of participants means that the results may not be generalizable. The small sample size also means that the statistics are generally weak, particularly in determining the linear relationship between age and burnout, or duration of work and burnout among healthcare professionals. The staffing structure used for sample size determination was a pre-COVID-19 structure and did not reflect the changes in staffing due to the COVID-19 pandemic. The study was a web-based survey, which could have excluded some of the potential participants, who may have had difficulties with internet connection and access. Additionally, the lack of a qualitative component in the survey did not allow the respondents to expound on their experiences of burnout. This limited the study’s in-depth exploration of the factors contributing to burnout among the staff at FPRRH.

In conclusion, burnout is prevalent across all carders and age groups among health professionals at FPRRH although the observed linear relationship between the age of healthcare professionals, their duration of work at the hospital and burnout score is not statistically significant. Moderate burn is the most prevalent, and the major associated factors are imbalances in duty allocation, physically exhausting work, and resource limitations. Most of the possible causes of burnout identified are within the scope of hospital leadership to address. The possible impact on staff performance and patient clinical outcomes is speculative, and additional studies are required. A larger possibly mixed-methods study is recommended to provide more insight on the qualitative component of the factors that contribute to burnout.

To mitigate the potential impact on patient care, measures to address the causes of burnout should be developed and implemented at the hospital. This may include formulating and availing platforms for healthcare staff to express concerns about work-related stress and devising means to address such concerns in a supportive environment where mental health conversations are encouraged and not stigmatised^26^. Such measures should be formulated in consultation with the staff who are the beneficiaries, as this may improve their utilization. Additionally, healthcare managers may include mandatory periodic breaks for all staff in the governing policies of healthcare institutions, and lead by example in fostering a culture of openness, empathy and support, to reduce stigma associated with mental health issues among healthcare professionals^18^. Mechanisms to address staff absenteeism also need to be enforced to minimize unfair duty coverage. For example, work monitoring tools such as cameras and computer software in addition to financial incentives like bonuses or fines according to attendance have been found to reduce staff absenteeism by 21 percentage points relative to the nonmonitored groups^55^.

### Supplementary information

Supplementry information

## Data Availability

The datasets used and analyzed during this study are available from the corresponding author upon reasonable request.
