# Supplementary material for: Prevalence of burnout among healthcare professionals: a survey at fort portal regional referral hospital
Source: Npj Ment Health Res. 2024 May 6;3:16. doi: 10.1038/s44184-024-00061-2 (PMC11074248; doi:10.1038/s44184-024-00061-2)
Supplement: Supplementary file 1 — Supplementry information [file 44184_2024_61_MOESM1_ESM.pdf]

PREVALENCE OF BURNOUT AMONG HEALTH CARE PROFESSIONALS: A SURVEY AT FORTPORTAL REGIONAL REFERRAL HOSPITAL

You're invited to participate in the research study: PREVALENCE OF BURNOUT AMONG HEALTH CARE PROFESSIONALS: A SURVEY AT FORTPORTAL REGIONAL REFERRAL HOSPITAL .

Kindly complete all sections of the questionnaire as truthfully as possible. Your participation in the study is voluntary and can be retrieved at any time with no consequences . Thank you for participating.

* Indicates required question

PARTICIPANT INFORMATION AND CONSENT

Principal investigator: Ian Batanda
Email address: ibatanda8@gmail.com
Contact number: +256 783 179 133

INTRODUCTION:
You are invited to participate in the research study stated above.

Participation in the study is entirely voluntary, and you are free to withdraw at any time. You will be requested to complete a self-administered questionnaire. This is expected to last approximately 5-10 minutes. Please answer all questions as truthfully as possible. All information provided will be used only for this study and will be treated with confidentiality.

RISK AND DISCONFORT
There is no anticipated physical risk to the study participants. However, some participants may be uncomfortable expressing details about their work. To minimize psychological distress, participants are reassured that participation or withdrawal from the study bears no consequences to them.

BENEFITS FOR PARTICIPATION
The study may not benefit the participant directly. There is no monetary benefit for participation in this study.
However, the results will shed light on the issues surrounding burnout among health professionals. The study will highlight the magnitude of the problem and present a basis for additional research into work-related stress among health care professionals. This is expected to inform policy regarding implementing measures to address workplace stress. It is also expected to highlight the growing need for health care professionals to actively seek mental health support and to improve staff welfare.

PAYMENT FOR PARTICIPATION
No remuneration will be given to participants in this research.

CONFIDENTIALITY
Throughout the process, participants will be identified by a code number and not their names or identifying information. All information collected will remain confidential and will be kept securely, to be accessed only by the researcher.

PARTICIPANT WITHDRAWAL
You may choose to participate in this study or not. If you choose to participate, you are free to withdraw at any time with no consequence.

QUESTIONS AND CONCERNS
In case of additional questions, the participant can contact the researcher at mobile phone number 0783179133. Email: ibatanda8@gmail.com

PARTRICIPANT RIGHTS
Participants with concerns about their rights as research participants can have their queries addressed by contacting Fort portal R.R Hospital administration on telephone number;

+25648322761. Email: fphosp@gmail.com.

Study approval

The study was approved by the FPRRH research and ethics committee, and the FPRRH administration.

BY CLICKING YES BELOW, YOU CONSENT TO PARTICIPATE IN THE STUDY UNDER THE CONDITIONS DESCRIBED ABOVE.

1. I agree to participate in the study under the conditions described above. *

Mark only one oval.

- Yes, I agree to participate
- No, I do not wish to participate

Demographic information

2. Gender *

Mark only one oval.

- Male
- Female

3. Age range in years *

Mark only one oval.

- 20-29
- 30-39
- 40-49
- 50-59

4. Duration of work at FPRRH *

Mark only one oval.

- 1 to 3 years
- 4 to 6 years
- 7 to 10 years
- More than 10 years

5. Employment category *

Mark only one oval.

- Doctor
- Nurse
- Allied health professional

Burnout survey

Please Select only one response.

6. Part one: Personal Burnout.

Mark only one oval per row.

	Always	Often	Sometimes	Seldom	Never
**How often do you feel tired?**	⬭	⬭	⬭	⬭	⬭
**How often are you physically exhausted?**	⬭	⬭	⬭	⬭	⬭
**How often are you emotionally exhausted**	⬭	⬭	⬭	⬭	⬭
**How often do you think: "I can't take it anymore"?**	⬭	⬭	⬭	⬭	⬭
**How often do you feel worn out because of your work?**	⬭	⬭	⬭	⬭	⬭
**How often do you feel weak and susceptible to illness?**	⬭	⬭	⬭	⬭	⬭

7. Part two: Work-related Burnout

Mark only one oval per row.

	To a very high degree	To a high degree	Somewhat	To a low degree	To a very low degree
**Is your work emotionally exhausting?**	◯	◯	◯	◯	◯
**Do you feel burnt out because of your work?**	◯	◯	◯	◯	◯
**Does your work frustrate you?**	◯	◯	◯	◯	◯
**Does duty allocation on your ward frustrate you?**	◯	◯	◯	◯	◯
**Is your work physically exhausting**	◯	◯	◯	◯	◯

8.

Mark only one oval per row.

	Always	Often	Sometimes	Seldom	Never
**Do you feel worn out at the end of the working day?**	◯	◯	◯	◯	◯
**Are you exhausted in the morning at the thought of another day at work?**	◯	◯	◯	◯	◯
**Do you feel that every working hour is tiring for you?**	◯	◯	◯	◯	◯
**Do you feel like your work duties are unfairly allocated?**	◯	◯	◯	◯	◯
**Does duty allocation on your ward need to change for you to enjoy time at work?**	◯	◯	◯	◯	◯
**Are you sometimes blamed for other**	◯	◯	◯	◯	◯

people's mistakes?					
Do you have enough energy for family and friends during leisure time?	⬭	⬭	⬭	⬭	⬭

9. Part three: client-related Burnout

Mark only one oval per row.

	To a very high degree	To a high degree	Somewhat	To a low degree	To a very low degree
**Do you find it hard to work with patients?**	⬭	⬭	⬭	⬭	⬭
**Do you find it frustrating to work with patients?**	⬭	⬭	⬭	⬭	⬭
**Does it drain your energy to work with clients?**	⬭	⬭	⬭	⬭	⬭
**Do you get emotionally attached to patients?**	⬭	⬭	⬭	⬭	⬭
**Do you get frustrated when you find there isn't much you can do for a patient?**	⬭	⬭	⬭	⬭	⬭

10.

Mark only one oval per row.

	Always	Often	Sometimes	Seldom	Never
**Do you feel that you give more than you get back when you work with patients?**	⬭	⬭	⬭	⬭	⬭
**Are you tired of working with patients?**	⬭	⬭	⬭	⬭	⬭
**Do you sometimes wonder how long you will be able to continue working with patients?**	⬭	⬭	⬭	⬭	⬭

This content is neither created nor endorsed by Google.

Google Forms
